# Impact of uncomplicated traumatic dental injuries on the quality of life of children and adolescents: a systematic review and meta-analysis

**DOI:** 10.1186/s12903-019-0916-0

**Published:** 2019-10-22

**Authors:** Diego Lopez, Nilakshi Waidyatillake, Carlos Zaror, Rodrigo Mariño

**Affiliations:** 1 Melbourne School of Population and Global Health, The University of Melbourne, Melbourne, VIC Australia; 2 Allergy and Lung Health Unit, Melbourne, School of Population and Global Health, The University of Melbourne, Melbourne, VIC Australia; 3 Department of Pediatric Dentistry and Orthodontics, Center for Research in Epidemiology, Economics and Oral Public Health (CIEESPO), Faculty of Dentistry, Universidad de La Frontera, Temuco, Chile; 4 Melbourne Dental School, University of Melbourne, Melbourne, Australia

**Keywords:** Uncomplicated, Traumatic dental injuries, Quality of life, OHRQoL, Children, Adolescents

## Abstract

**Background:**

Traumatic dental injuries (TDIs) are highly prevalent during childhood and adolescence and have a significant effect on their oral health related quality of life (OHRQoL). Uncomplicated TDIs, dental trauma involving enamel, enamel and dentin and tooth discolorations, account for approximately two-thirds of all diagnosed TDIs in children and adolescents. Hence, it may be important to understand the impact of uncomplicated TDIs on OHRQoL, by synthesizing the available literature.

**Methods:**

Medline, Embase, Web of Science and Scopus databases were systematically searched from January 1966 to April 2018. Studies that evaluated the effect of TDIs on the OHRQoL of children and adolescents using validated methods were selected for analysis. A narrative synthesis and a meta-analysis were performed. The studies were pooled according to age groups and OHRQoL questionnaire used. A random-effect model was applied to calculate the pooled odds ratios (OR) and their respective 95% confidence intervals.

**Results:**

There were 712 identified studies. Of these, 26 articles were selected for the review and included in the narrative synthesis, 20 of these articles concluded that uncomplicated TDIs were not associated with a negative impact in OHRQoL. Seventeen were included in the meta-analysis. The estimates were pooled by age groups: children (OR: 1.01; 95%CI; 0.85–1.19; I^2^ = 51.9%) and adolescents (OR: 1.07; 95%CI; 0.91, 1.26; I^2^ = 50.2%).When pooling all estimates the OR was 0.96 (95% CI: 0.85–1.10; I^2^ = 61.4%).

**Conclusions:**

Uncomplicated TDIs do not have a negative impact on the OHRQoL of children and adolescents. Further prospective studies are needed to confirm the results of this review. The majority of the studies included were of cross-sectional design, which may have limited the ability to reach conclusions on the nature of this association. The PROSPERO systematic review registry is CRD42018110471.

## Background

Traumatic dental injuries (TDIs) are one of the most prevalent oral pathologies in children and adolescents [1]. Approximately 22.7% of children aged 0 to 6 years have a TDI involving the primary dentition in their early childhood [1] and nearly 25% of all schoolchildren and adolescents aged 7 to 19 years have a TDI involving their permanent dentition [2, 3]. Uncomplicated TDIs (enamel fractures, enamel and dentin fractures and tooth discolorations) are the least severe, but most frequent type of TDI in children and adolescents, as they account for approximately two thirds of all diagnosed TDIs [2].

Previous studies showed that subsequent to the dental tissue trauma (e.g., enamel or dentinal fracture), bacterial invasion in the fracture could determine further infection and exposed dentinal tubules can cause pulp inflammation that leads to either repair or necrosis of the pulp [3]. Discoloration of the tooth after trauma can negatively affect the Oral Health related Quality of Life (OHRQoL) of children and adolescents [4, 5]. Also, the monetary costs of immediate and follow-up care to dental trauma patients, their families and oral health services are substantial [6]. In this way, uncomplicated TDIs, can have medium and long-term consequences, which should be identified, monitored and their treatment needs assessed [7]. Thus, clarification of the effects and consequences of uncomplicated TDIs on the quality of life of adolescents and children would be beneficial for the public health care system.

In recent times, researchers are increasingly using OHRQoL measures to evaluate the effect that oral conditions such as TDIs have on quality of life. OHRQoL is a measure of the impact of oral conditions on daily functioning; for example the impact oral conditions have on a patient’s wellbeing when talking, smiling, laughing, sleeping, and eating; their satisfaction and; their self-esteem [8]. A series of validated questionnaires which are self-reported or answered by proxy are used to measure OHRQoL [9]. Some examples of these questionnaires for children include the Early Childhood Oral Health Impact Scale (ECOHIS) [10] which uses parents or guardians as proxy and the Scale of Oral Health Outcomes for 5-year-olds (SOHO-5) [11] which is self-reported. And for adolescents, the child perceptions questionnaire (CPQ) for ages 11–14, [12], with a version for younger children aged 8–10 which are self-reported [13].

Two previous systematic reviews quantified the effect of TDIs, without considering the type of TDIs, on the OHRQoL of preschool children and schoolchildren [14, 15]. Both systematic reviews concluded that TDIs have a negative impact on the OHRQoL of preschool children and schoolchildren. However, neither of these reviews explored the implications of only uncomplicated TDIs on the OHRQoL of children and adolescent. Given the frequency of this type of traumatic dental injury, it is worthwhile to determine its consequences on quality of life. Therefore, the research question is as it follows: Do uncomplicated TDIs affect the OHRQoL of children and adolescents? And the aim is to evaluate the impact of uncomplicated TDIs on the OHRQoL of children and adolescents, by synthesizing the available literature.

## Methods

This review was prospectively registered in PROSPERO (CRD42018110471) and follows the Guidelines of the Preferred Reporting Items for Systematic Reviews and Meta-Analysis (PRISMA) [16].

### Search strategy and eligibility criteria

A systematic search of literature was done on Medline, Embase, Web of Science and Scopus, from January 1966 to April 2018. Using MeSH terms and keywords related to dental traumatic injuries and quality of life the databases were systematically searched. The search terms used in Medline and EMBASE ovid were: (Dental or tooth or teeth) AND adj5 (Trauma* or injur*) AND Child* or infant* or adolescen* or toddler* or young* or minor* AND quality of life. Additional articles were identified from the reference section of the selected studies from the original search. Further details of the search terms are provided in Additional file 1.

The inclusion criteria were as follows: Studies with epidemiological design (case-control, cross-sectional, and cohort) in English, Spanish and Portuguese; traumatic dental injuries as the exposure variable and was measured with an established criteria for diagnosis; quality of life as the outcome and measured using a validated questionnaire [12, 13, 17].; presented the type of traumatic dental injuries group stratification; study samples that includes children and/or adolescents up to 19 years of age [18]. The exclusion criteria were as follows: case reports, studies and articles without the predefined outcome and exposure; studies that focus on particular groups (athletes, patients with cerebral palsy, and victims of violence).

### Study selection and data extraction

Endnote™ X8.2 reference management software for Windows (9 January 2018 release) was used to manage relevant citations. A database was created to facilitate management and checked for duplicate articles. Two researchers (DL and CZ) independently reviewed the titles and abstracts. Discrepancies were resolved by consulting a third reviewer (RM). Citations were sorted based on the relevance of the articles to the research question and selection criteria into: “not relevant”, “relevant”, “highly relevant” and “final selection”. Full text of the “highly relevant” group were reviewed. Studies which met the requirements for the qualitative and/or quantitative synthesis were placed into the “final selection” group. Neither authors nor journals were blinded to reviewers.

Data extraction of the studies were conducted by agreement of two reviewers (DL and CZ) using a predefined, standardized data collection form. Using four potentially eligible articles a pilot test was performed to homogenize criteria among reviewers. The Kappa statistic was used (K = 0.79), which demonstrated substantial agreement between the reviewers. The data extracted from the selected studies were: Author and year, Title, country, type of population and sample size, exposure, age when was measured and its definition, outcome, age when was measured and its definition, Stratification, confounders/selection bias, reverse causation, results and findings by the authors. The effect estimate is the dichotomous association between uncomplicated TDI and OHRQoL of children and adolescents, and this was extracted from each paper. Uncomplicated TDIs were classified as those in which dislocation of the tooth and/or pulp tissue was not involved; that is, enamel fractures, enamel and dentin fractures, and tooth discolorations. Complicated TDIs were classified as those in which dislocation of the tooth and/or pulp was involved. We contacted study authors by e-mail to obtain additional information when data were missing or unclear.

### Quality assessment

Two authors were independently involved in the quality assessment of the studies methodological quality (DL and CZ). Using the Newcastle-Ottawa scale (NOS) adapted for observational studies, [19] a star system was developed to assess study quality. Each study is judged on three broad perspectives: the ascertainment of either the exposure or outcome: the comparability of the groups; and the selection of the study groups [19]. There are no universally accepted standardized grading methods for NOS, the quality of the studies were graded as follows: very good (8–9), good (6–7), satisfactory (5–4) and unsatisfactory (0–3). The scale is provided in Additional file 2.

### Data analysis

Following Green et al. recommendations [20], the narrative overview model which is broad narrative syntheses of formerly published studies, was used. The full text of the selected studies was examined and information on these was extracted into tables. The studies were divided by study design groups and study characteristics within each group were reported to articulate broader similarities and differences among and between the groups.

The main outcome was the impact on OHRQoL measured through validated OHRQoL instruments. Studies that provided the effect estimates Odds ratio (OR) or the data necessary for calculating the effect estimate were included in the meta-analysis. The later was used to generate single 2 by 2 tables and the odds ratios and their respective 95% confidence intervals were calculated for each study. Subsequently, random effects meta-analysis was performed and studies were grouped according to age groups and OHRQoL questionnaire used. The I^2^ was used to evaluate the heterogeneity of the pooled Odds ratio (25, 50, and 75% were considered to be low, moderate and high heterogeneity respectively) [21]. The decision of pooling the studies by age groups and OHRQoL questionnaire used was done a priori because it is expected to be a source of heterogeneity. Data were not pooled if I^2^ was over 75%.

In addition, Funnel plots and correspondent Egger’s test were done to explore possible publication bias. All analysis were performed using Stata Statistical Software: Release 13 (Stata Corp, College Station, TX, USA).

## Results

**Fig. 1:**
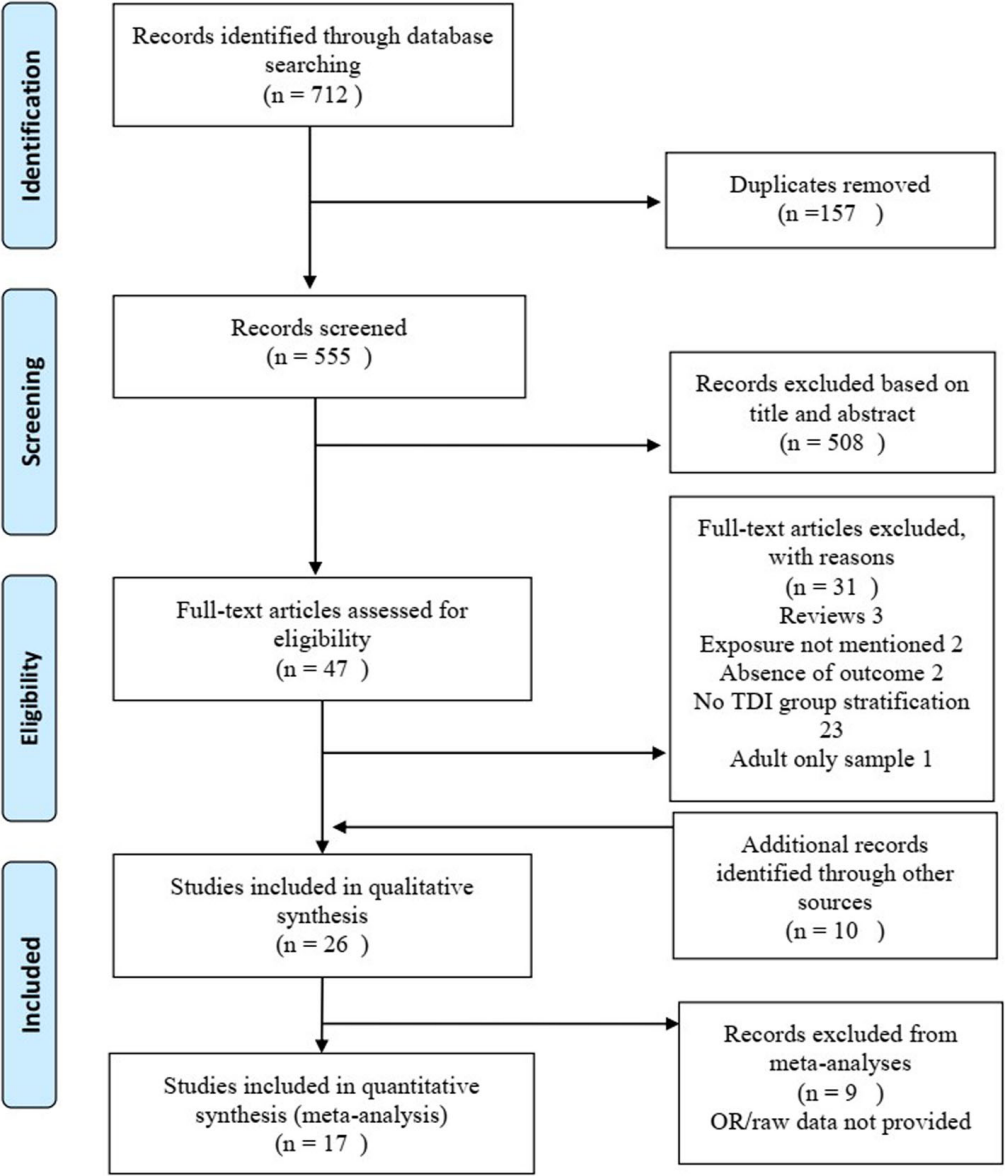
PRISMA flowchart of the selection of eligible literature

The PRISMA diagram (Fig. 1) shows the selection of studies in this review. Thorough electronic searches identified 712 studies. After excluding 157 duplicates and after reviewing titles and abstracts, 47 publications were evaluated in full text. Of those, 31 studies were excluded and ten additional studies were identified from the reference section of the studies selected for full text review, giving a total of 26 studies, 25 studies in English and one study in Portuguese. The characteristics of these studies are presented in Table 1. The list of excluded studies after the full-text review is provided in Additional file 3.

**Table 1:** Characteristics of included studies

Study	Study design	Country	Analysed sample/total sample	Setting	Diagnostic criteria	OHRQoL questionnaire (age)	Effect estimates measure
Soares, Barasuol et al. 2018 [22]	Cross-sectional	Brazil	1589/1671	Population-based	Andreasen classification	CPQ8–10 (8–10 years)	Prevalence ratio
Silva-Oliveira, Goursand et al. 2018 [23]	Cross-sectional	Brazil	588/633	Population-based	Andreasen classification	CPQ11–14 (12 years)	Odds ratio
Martins, Sardenberg et al. 2018 [24]	Cross-sectional	Brazil	1204/1439	Population-based	Andreasen classification	CPQ8–10 (8–10 years)	Odds ratio
Ramos-Jorge, Sa-Pinto et al. 2017 [4]	Cross-sectional	Brazil	391/459	preschool-based	Andreasen classification	ECOHIS (3–5 years)	Prevalence ratio
Neves, Perazzo et al. 2017 [25]	Cross-sectional	Brazil	769/769	school-based	Andreasen classification	SOHO-5 (5 years)	Prevalence ratio
Gonçalves, Dias et al. 2017 [26]	Cross-sectional	Brazil	192/192	school-based	Andreasen classification	ECOHIS (3–5 years)	Risk ratio
Gomes, Perazzo et al. 2017 [27]	Cross-sectional	Brazil	769/769	school-based	Andreasen classification	SOHO-5 (5 years)	Prevalence ratio
Bomfim, Herrera et al. 2017 [28]	Cross-sectional	Brazil	7328/7328	Population-based	Andreasen classification	OIDP (12 years)	Odds ratio
Pulache, Abanto et al. 2016 [29]	Cross-sectional	Peru	473/513	school-based	Andreasen classification	CPQ11–14 (11–14 years)	Risk ratio
Firmino, Gomes et al. 2016 [30]	Case-control	Brazil	830/845	Population-based	Andreasen classification	ECOHIS (3–5 years)	Odds ratio
Feldens, Day et al. 2016 [31]	Cross-sectional	Brazil	1683 /1683	Population-based	Andreasen classification	ECOHIS (3–5 years)	Prevalence ratio
Vieira-Andrade, Siqueira et al. 2015 [32]	Case-control	Brazil	335/335	Population-based	Andreasen classification	ECOHIS (3–5 years)	Odds ratio
Freire-Maia, Auad et al. 2015 [33]	Cross-sectional	Brazil	1201/1201	school-based	Andreasen classification	CPQ8–10 (8–10 years)	Odds ratio
Abanto, Tello et al. 2015 [34]	Cross-sectional	Brazil	1215/1215	Population-based	Glendor classification	ECOHIS (1–4 years)	Prevalence ratio
Viegas, Paiva et al. 2014 [5]	Cross-sectional	Brazil	1632/1632	School-based	Andreasen classification	ECOHIS (5–6 years)	Odds ratio
Gomes, Pinto-Sarmento et al. 2014 [35]	Cross-sectional	Brazil	834/864	preschool-based	Andreasen classification	ECOHIS (3–5 years)	Odds ratio
Bendo, Paiva et al. 2014 [36]	Case-control	Brazil	1215/1215	Population-based	Andreasen classification	CPQ11–14 (11–14 years)	Odds ratio
Abanto, Tsakos et al. 2014 [37]	Cross-sectional	Brazil	335/394	Dental school-based	Glendor classification	SOHO-5 (5–6 years)	Risk ratio
Siqueira, Firmino et al. 2013 [38]	Cross-sectional	Brazil	814/864	Population-based	Andreasen classification	ECOHIS (3–5 years)	Odds ratio
Dame-Teixeira, Alves et al. 2013 [39]	Cross-sectional	Brazil	1528/1837	School-based	O’Brien classification	CPQ11–14 (14 years)	Risk ratio
Viegas, Scarpelli et al. 2012 [40]	Cross-sectional	Brazil	388 /413	preschool-based	Andreasen classification	ECOHIS (5 years)	Risk ratio
Traebert, de Lacerda et al. 2012 [41]	Cross-sectional	Brazil	403/409	Population-based	WHO criteria	CPQ11–14 (11–14 years)	Prevalence ratio
Piovesan, Abella et al. 2011 [42]	Cross-sectional	Brazil	713/ 792	School-based	O’Brien classification	CPQ11–14 (12 years)	Risk ratio
Aldrigui, Abanto et al. 2011 [43]	Cross-sectional	Brazil	260/305	preschool-based	Andreasen classification	ECOHIS (2–5 years)	Risk ratio
Bendo, Paiva et al. 2010 [44]	Cross-sectional	Brazil	1612 /1870	School-based	Andreasen classification	CPQ11–14 (11–14 years)	Prevalence ratio
Piovesan, Antunes et al. 2010 [45]	Cross-sectional	Brazil	713/ 792	School-based	O’Brien classification	CPQ11–14 (11–14 years)	Risk Ratio

*OHRQoL* Oral health related quality of life, *CPQ8–10* Child Perceptions Questionnaire version for 8 to 10 years old, *CPQ11–14* Child Perceptions Questionnaire version for 11 to 14 years old, *ECOHIS* Early Childhood Oral Health Impact Scale, *SOHO-5* Scale of Oral Health Outcomes for Five-Year-Old Children

### Narrative synthesis

The 26 papers included in the review were grouped by study design: 3 were case control studies [30, 32, 36] and there were 23 cross-sectional studies [4, 5, 22–29, 31, 33–35, 37–45]. The study findings were reported in condensed tables that summarized the information extracted from each paper. This table is provided in Additional file 4.

The Andreasen Classification [3] was used in 19 studies. Others used either Glendor’s [46] or O’Brien’s classifications [47]. The age of participants also differed between studies; children (1 to 6 years), adolescents (8 to 14 years). All studies used trained and calibrated dentists to diagnose the TDIs. TDIs were presented by type of injury and some studies presented TDIs as either complicated or uncomplicated.

The use of OHRQOL instruments was according to age of participants. For adolescent samples, 3 studies used the Child Perceptions Questionnaire (CPQ) version for 8 to 10 years (CPQ 8–10) [22, 24, 33], 8 studies used the CPQ version 11 to 14 years (CPQ 11–14) [23, 29, 36, 39, 41, 42, 44, 45] and 1 study used the Oral Impact on Daily Performance (OIDP) [28]. From the studies that used samples of children, 11 studies used the Early Childhood Oral Health Impact Scale (ECOHIS) [4, 5, 26, 30–32, 34, 35, 38, 40, 43] and 3 studies used the Scale of Oral Health Outcomes for Five-Year-Old Children (SOHO-5) [25, 27, 37].

The majority of the cross-sectional studies (*n* = 15) determined that uncomplicated TDIs were not associated with a negative impact on OHRQoL [5, 24–27, 29, 34, 35, 37, 38, 40, 42–45]. However, 4 studies found an association between uncomplicated TDIs and negative impact on quality of life [23, 33, 39, 41]. Nevertheless, these studies presented estimates with wide confidence intervals and high *p*-values, which reduced their statistical significance. The remaining 4 cross-sectional studies explored the effect of either only enamel fractures and/or enamel and dentin fractures in OHRQoL [4, 22, 28, 31]. Remarkably, these papers concluded that only enamel fractures did not have a negative effect on the OHRQoL. Yet, enamel and dentin fractures were associated with a negative impact on the oral health related quality of life of children and adolescents.

### Study quality

All of the studies included in the review were observational studies. Table 2 shows that all three case-control studies were considered ‘Very good’ quality (i.e. score of 8 or more). Similarly, most of the cross-sectional studies achieved 8 or more out of 9. However, 4 achieved 6 or 7 and two studies scored 5, which is considered ‘Good’ and ‘Satisfactory’, respectively. Common areas of strength were the ascertainment of the exposure and the assessment of the outcome because the sample was randomly selected from schools in most studies. Also, all of the studies used established diagnosis for TDIs and validated instruments to assess OHRQoL. These were measured by trained and calibrated examiners. Hence, the studies included presented low risk of bias.

**Table 2:** Study quality assessment

Study	Selection criteria				Comparability	Outcome			
	Case definition	Representativeness of the cases	Selection of controls	Definition of controls		Ascertainment of exposure	Same method of ascertainment for cases and controls	Non-respondent rate	Total score
Case-control Studies									
Firmino et al. 2016 [30]	★	★	★	★	★	★	★	★	8/9
Vieira-Andrade et al. 2015 [32]	★	★	★	★	★★	★	★	★	9/9
Bendo et al. 2014 [36]	★	★	★	★	★★	★	★	★	9/9
Cross-sectional studies									
Soares et al. 2018 [22]	★	★	★	★	★★	★	★	★	9/9
Silva-Oliveira et al. 2018 [23]			★	★	★★	★	★	★	7/9
Martins et al. 2018 [24]	★	★	★		★★	★	★	★	9/9
Ramos-Jorge et al. 2017 [4]		★	★	★	★★	★	★	★	8/9
Neves et al. 2017 [25]	★	★	★	★	★★	★	★	★	9/9
Gonçalves et al. 2017 [26]		★	★		★	★		★	5/9
Gomes et al. 2017 [27]	★	★	★	★		★	★	★	7/9
Bomfim et al. 2017 [28]	★	★	★	★	★	★	★	★	8/9
Pulache et al. 2016 [29]		★	★	★	★★	★	★	★	8/9
Feldens et al. 2016 [31]	★	★	★	★	★★	★	★	★	9/9
Freire-Maia et al. 2015 [33]	★	★	★	★	★★	★	★	★	9/9
Abanto et al. 2015 [34]	★	★	★	★	★★	★	★	★	9/9
Viegas et al. 2014 [5]	★	★	★	★	★★	★	★	★	9/9
Gomes et al. 2014 [35]	★	★	★	★	★★	★	★	★	9/9
Abanto et al. 2014 [37]		★	★	★	★★	★	★	★	8/9
Siqueira et al. 2013 [38]	★	★	★	★	★★	★	★	★	9/9
Dame-Teixeira et al. 2013 [39]	★	★	★		★	★	★	★	7/9
Viegas et al. 2012 [40]		★	★		★★	★	★	★	7/9
Traebert et al. 2012 [41]		★	★	★	★★	★	★	★	8/9
Piovesan et al. 2011 [42]	★	★	★	★	★★	★	★	★	9/9
Aldrigui et al. 2011 [43]			★		★★		★	★	5/9
Bendo et al. 2010 [44]	★	★	★		★★	★	★	★	8/9
Piovesan et al. 2010 [45]	★	★	★	★	★★	★	★	★	9/9

Based on the Newcastle-Ottawa scale (NOS) adapted for observational studies

Of the studies included, 21 adjusted for oral conditions (dental caries and/or malocclusion) and other confounding factors such as sex, age, socio-economic status. The remainder presented unadjusted estimates, only. The overwhelming majority (*n* = 20), concluded that uncomplicated TDIs were not associated with a negative impact on the quality of life of children and adolescents. Two of the three case control studies did not find evidence that uncomplicated TDI affected OHRQoL in pre-schoolers (OR: 1.05; 95% CI: 0.54–1.99) [32] and adolescents (OR: 0.64; 95% CI: 0.38–1.06 [36]. The third case control study [30] found that pre-schoolers with uncomplicated TDIs had greater odds of having their OHRQoL impacted compared to children without TDIs (OR: 1.57; CI: 0.92–2.64).

### Quantitative analysis

**Fig. 2:**
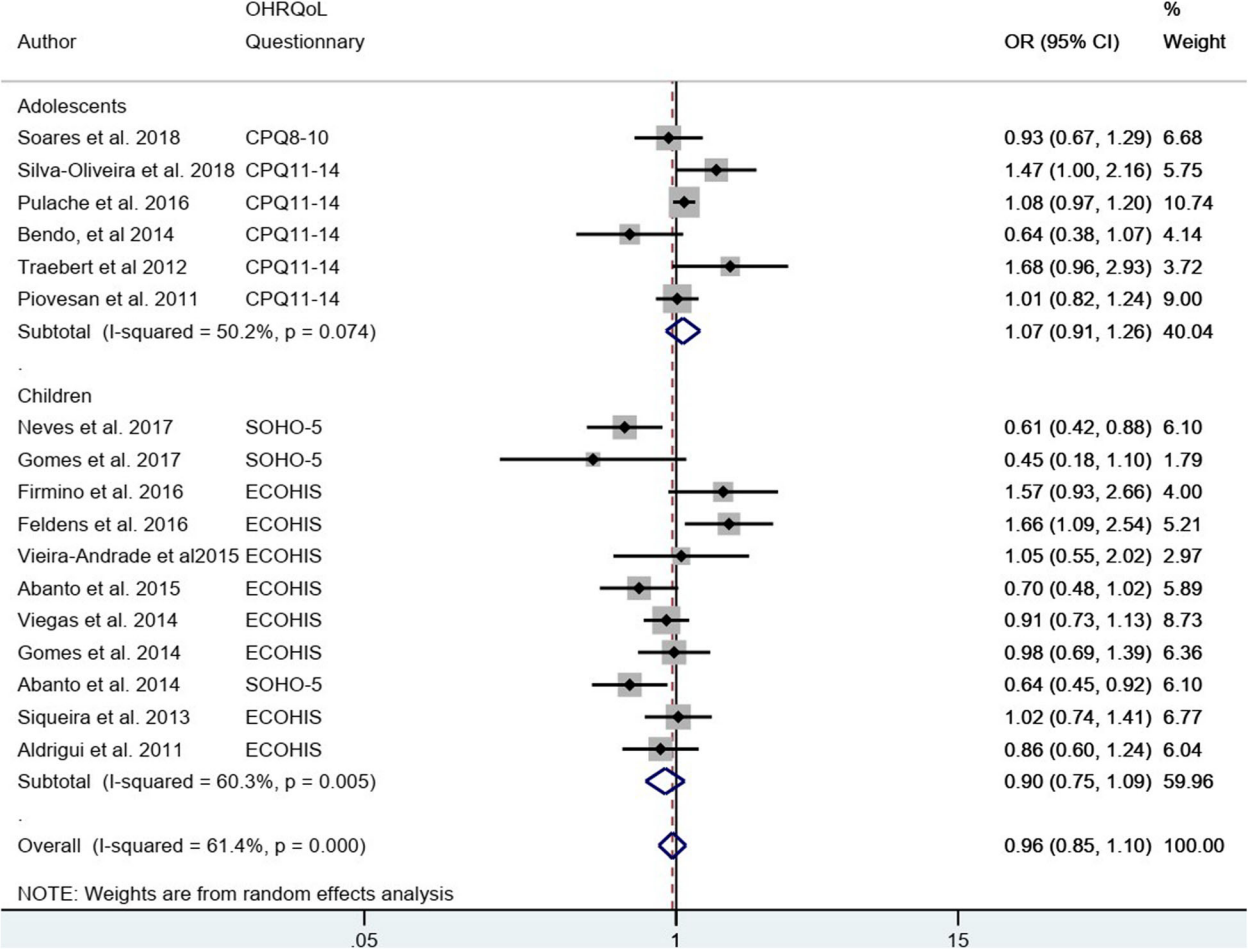
Meta-analysis: The association of uncomplicated TDI and OHRQoL by age groups

**Fig. 3:**
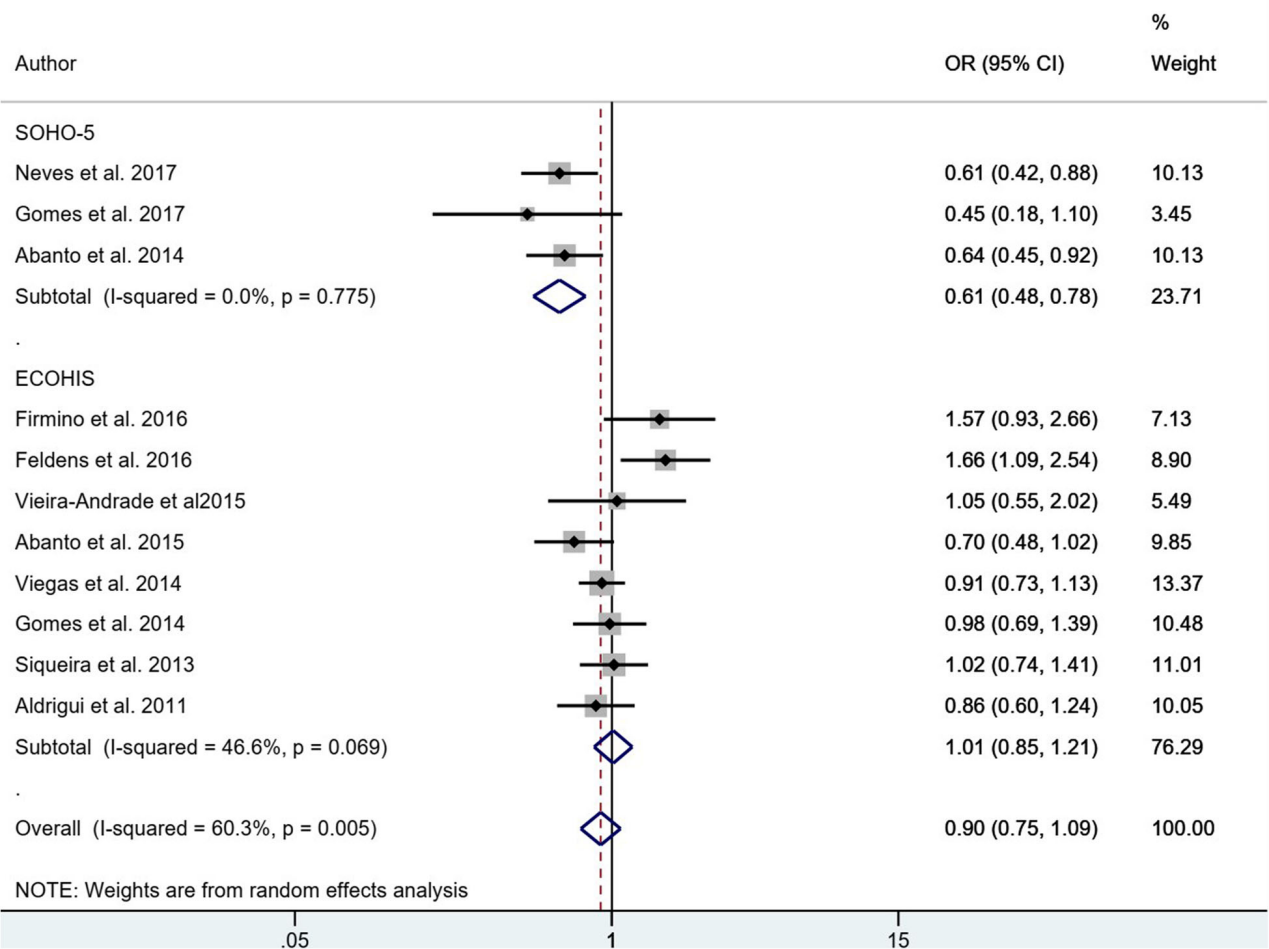
Meta-analysis: The association of uncomplicated TDI and OHRQoL by OHRQoL questionnaire used in children studies

Nine studies were excluded from the meta-analysis because they did not provide the Odds ratio or the necessary data for its estimation [4, 24, 26, 28, 33, 39, 40, 44, 45]. The 17 studies included in quantitative analysis had 14,457 participants, which were evaluated for the dichotomous association between uncomplicated TDIs (presence/absence) and any impact on OHRQoL (presence/absence) (Fig. 2). The sub-total estimates for age groups (in two groups: early children (4 to 6 years old) and adolescents (8 to 19 years old)) are different: the pooled OR for studies of children was 0.90 (95% CI: 0.75–1.09), while heterogeneity was considerable (I^2^ = 60.3%) compared to adolescent studies with pooled OR 1.07 (95% CI: 0.91–1.26) and moderate heterogeneity (I^2^ = 50.2%). Both estimated confidence intervals included the null value (i.e.: 1), the overall pooled OR was 0.96 (95% CI: 0.85–1.10) and heterogeneity was moderate (I^2^ = 61.4%). The adolescent studies used the same OHRQoL questionnaire. However, the children studies used 2 types of OHRQoL questionnaire, for that reason a separate meta-analysis is presented. (Fig. 3) The sub-total estimates are different: SOHO-5 with a pooled OR of 0.61 (95% CI: 0.48, 0.78) and ECOHIS with a pooled OR of 1.01 (95% CI: 0.75, 1.09). The dataset is available in Additional file 5.

**Fig. 4:**
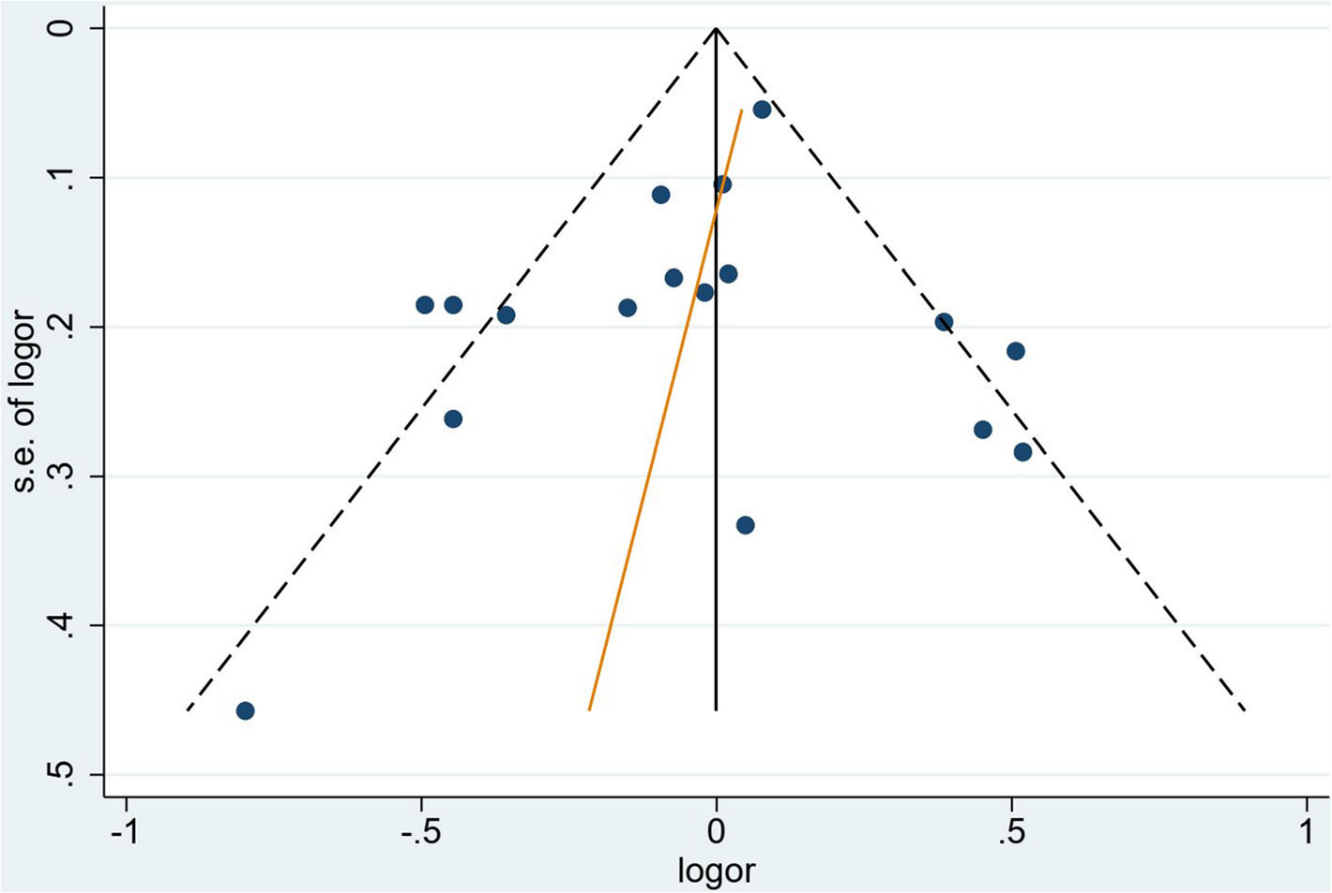
Funnel plot with correspondent Egger’s test for all included studies. (*p*: 0.415)

The risk of publication bias for the 17 studies included in the meta-analysis was assessed using a funnel plot and the Egger’s test (p: 0.415) in Fig. 4. The funnel is approximately symmetrical and the Egger’s test failed to detect publication bias. This implies that publication bias does not seems to be significant to the validity of the research.

## Discussion

The narrative synthesis and the meta-analysis provided evidence that children and adolescents with uncomplicated TDIs have similar chances of any impact on OHRQoL to children and adolescents without TDIs. Most of the studies included in the review concluded that uncomplicated TDIs were not associated with a negative impact on the OHRQoL of life of children or adolescents. This is reflected in the findings of the meta-analysis, even after adjusting by age groups. Concordantly, some authors have suggested that enamel fractures, which are the most common uncomplicated TDI, have a minimal impact on OHRQoL from the standpoint of children and their families [4, 22, 24, 31, 42].

This review focused on the consequences of uncomplicated traumatic dental injuries alone as opposed to the implications of all types of TDIs combined, as in the two previously published systematic reviews [14, 15]. Present findings differ from these two systematic reviews. This may be due to differences in the research question, and because the estimates represent the effect of the less severe type of TDI on the OHRQoL of children and adolescents. One of these reviews [15] concluded that complicated TDIs were associated with a higher OHRQoL impact relative to uncomplicated TDIs.

As shown by the exposure assessment in the narrative synthesis, there is no universally accepted set of diagnostic, classification, and registration criteria for TDIs, which makes true comparisons difficult [48]. As argued by Petti et al., the majority of TDIs are relatively easy to diagnose; however, their classification is difficult because there are more than 50 distinct diagnostic criteria, and on top of that, of course, more than one injury can happen at the same time [1]. Since the diagnosis of TDI was based on prevalent cases, some TDI fractures, such as crown fractures, could be misclassified with other dental condition such as tooth wear or developmental dental defects [49].

From the narrative synthesis, the outcome assessment revealed that for studies with preschooler samples, the most used validated questionnaire was the ECOHIS, which relies on a proxy (parents or guardians) to answer the questionnaire. A systematic review concluded that parents have limited knowledge and a different perspective about some aspects compared with their children, especially perspectives related to quality of life and its dimensions [50]. This could lead to parents or guardians underestimating the true impact of children’s oral conditions on their oral health related quality of life. Furthermore, oral trauma in primary teeth is often disregarded by parents and guardians, unless the TDI is severe [50].

Studies with adolescent samples used either the CPQ for 11 to 14 years or its 8 to 10 years version (CPQ 8–10). These instruments rely on self-reported answers, which may provide more precise data on how quality of life is affected by TDIs. The use of the same questionnaire can explain the moderate heterogeneity compared to studies with samples of children in the meta-analysis. This review noticed an age gap in the current literature evaluating the impact of TDIs on the OHRQoL of children age 6 to 8 years. In general, studies with samples of children presented lower effect estimates compared to studies with adolescent samples, for the association between uncomplicated TDIs and OHRQoL. This could be explained, firstly, because the ECOHIS relies on proxy answers from a parent or guardian and a large percentage of guardians or parents may not recognise the occurrence of minor TDIs in their children [50, 51]; and secondly, adolescents may be more capable of understanding TDI’s effects on their quality of life. Further research should clarify the clinical importance of this difference.

The heterogeneity for the 17 studies included in the meta-analysis was substantial (I^2^: 61.4%). The primary source of heterogeneity was the different age of the samples; for that reason, sub-group analysis by age group was performed to reduce the heterogeneity. Other sources of heterogeneity were the fact that some studies were school-based, clinic-based or population-based, and the use of different diagnostic criteria for TDIs. Conversely, an influence that decreased heterogeneity was the fact that almost all of the studies were from Brazil.

Some limitations to this study must be noted. The most obvious one is that the majority of the studies included were of cross-sectional design, which may have limited the ability to reach conclusions on the nature of this association. Therefore, effects over a period of time may not have been properly evaluated. Additionally, the prevalence of the impact on OHRQoL could be overestimated due the lowest cut-off point (at least one item) used. Also, the external validity of the study might be reduced given that almost all of the studies were from Brazil.

On the other hand, the use of the broad search strategy is one of the strengths of the study, as it ensures that all studies on this topic were included. Another strength is that it used both narrative synthesis and meta-analysis to assess the association between uncomplicated TDIs and OHRQoL. Additionally, the decision to include studies with an established diagnostic criteria and a validated questionnaire further reinforced the reliability of the data. Finally, the high quality of the studies included and the absence of publication bias increased the validity of present results.

## Conclusion

The narrative synthesis and the meta-analysis provided evidence that children and adolescents with any uncomplicated TDIs have similar chances of having an impact on OHRQoL to children and adolescents without TDIs. Suitable cut-off points to define when OHRQoL is impacted in children and adolescents are required. Public health programs can achieve the maximum impact in reducing the negative impact on OHRQoL from dental injury if more severe TDIs are the focus of such programs. However, the implications of uncomplicated TDIs over time is not well understood, and prospective cohort studies are required to confirm the results of this review.

## Supplementary information

**Additional file 1.** Specific search strategy: Includes the specific terms used in the literature search in the mentioned databases.

**Additional file 2.** The Newcastle-Ottawa Scale (NOS) for Assessing the Quality of case control studies and Cross-sectional: Study quality assessment tool used in the review.

**Additional file 3.** List of excluded studies and reasons after the full-text review: Studies excluded from the review with the reasons.

**Additional file 4.** Study characteristics and the association between uncomplicated TDI and OHRQoL in children and adolescents: Condensed tables that summarized the information extracted from each paper.

**Additional file 5.** Study dataset: The dataset used in the meta-analysis.

## Data Availability

All data generated or analysed during this study are included in this published article [and its supplementary information files].

## Abbreviations

CPQ 11–14: Child Perceptions Questionnaire version for 11 to 14 years old
CPQ 8–10: Child Perceptions Questionnaire version for 8 to 10 years old
ECOHIS: Early Childhood Oral Health Impact Scale
OHRQoL: Oral health related quality of life
SOHO-5: Scale of Oral Health Outcomes for Five-Year-Old Children
TDIs: Traumatic Dental Injuries
