# Supplementary material for: Impact of uncomplicated traumatic dental injuries on the quality of life of children and adolescents: a systematic review and meta-analysis
Source: BMC Oral Health. 2019 Oct 22;19:224. doi: 10.1186/s12903-019-0916-0 (PMC6805369; doi:10.1186/s12903-019-0916-0)
Supplement: Supplementary file 1 — Additional file 1. Specific search strategy: Includes the specific terms used in the literature search in the mentioned databases. [file 12903_2019_916_MOESM1_ESM.docx]

**Specific search strategy**

The search terms used in Medline and EMBASE ovid were: (Dental or tooth or teeth) AND adj5 (Trauma* or injur*) AND Child* or infant* or adolescen* or toddler* or young* or minor* AND quality of life.

The search terms for Web of science were: TS=(dental or tooth or teeth) AND TS= (Trauma* or injur*) AND TS=( Child* or infant* or adolescen* or toddler* or young* or minor*) AND TS=(quality of life).

Finally, the search terms for Scopus were: ( TITLE-ABS-KEY ( dental OR teeth OR tooth W/5 injur* OR trauma* ) ) AND ( quality AND of AND life ) AND ( child* OR toddler* OR minor* OR adolescent* OR young* ) AND ( LIMIT-TO ( SRCTYPE , "j " ) ) AND ( LIMIT-TO ( DOCTYPE , "ar " ) OR LIMIT-TO ( DOCTYPE , " re " ) ) AND ( LIMIT-TO ( SUBJAREA , "DENT " ) ) AND ( LIMIT-TO ( LANGUAGE , "English " ) OR LIMIT-TO ( LANGUAGE , " Portuguese " ) OR LIMIT-TO ( LANGUAGE , " Spanish " ) )
