# Supplementary material for: Impact of uncomplicated traumatic dental injuries on the quality of life of children and adolescents: a systematic review and meta-analysis
Source: BMC Oral Health. 2019 Oct 22;19:224. doi: 10.1186/s12903-019-0916-0 (PMC6805369; doi:10.1186/s12903-019-0916-0)
Supplement: Supplementary file 2 — Additional file 2. The Newcastle-Ottawa Scale (NOS) for Assessing the Quality of case control studies and Cross-sectional: Study quality assessment tool used in the review. [file 12903_2019_916_MOESM2_ESM.docx]

**The Newcastle-Ottawa Scale (NOS) for Assessing the Quality of case control studies and Cross-sectional.**

Note: A study can be awarded a maximum of one star for each numbered item within the Selection and Exposure categories. A maximum of two stars can be given for Comparability.

The studies that met at least five NOS criteria were considered to be studies with acceptable quality.

1. **Newcastle-Ottawa Quality Assessment Scale: Case Control/Cross-sectional**

Note: A study can be awarded a maximum of one star for each numbered item within the Selection and Exposure categories. A maximum of two stars can be given for Comparability.

**Selection**

1) Is the case definition adequate?

a) yes, with independent validation **(one star)**

b) yes, eg record linkage or based on self reports

c) no description

2) Representativeness of the cases

a) consecutive or obviously representative series of cases **(one star)**

b) potential for selection biases or not stated

3) Selection of Controls

a) community controls **(one star)**

b) hospital controls

c) no description

4) Definition of Controls

a) no history of disease (endpoint) **(one star)**

b) no description of source

**Comparability**

1) Comparability of cases and controls on the basis of the design or analysis

a) study controls for other oral conditions (dental caries, malocclusion) **(one star)**

b) study controls for socio and economic factors **(one star)**

**Exposure**

1) Ascertainment of exposure

a) secure record (eg surgical records) **(one star)**

b) structured interview where blind to case/control status **(one star)**

c) interview not blinded to case/control status

d) written self report or medical record only

e) no description

2) Same method of ascertainment for cases and controls

a) yes **(one star)**

b) no

3) Non-Response rate

a) same rate for both groups **(one star)**

b) non respondents described

c) rate different and no designation
