# Supplementary material for: Impact of uncomplicated traumatic dental injuries on the quality of life of children and adolescents: a systematic review and meta-analysis
Source: BMC Oral Health. 2019 Oct 22;19:224. doi: 10.1186/s12903-019-0916-0 (PMC6805369; doi:10.1186/s12903-019-0916-0)
Supplement: Supplementary file 3 — Additional file 3. List of excluded studies and reasons after the full-text review: Studies excluded from the review with the reasons. [file 12903_2019_916_MOESM3_ESM.docx]

**List of excluded studies and reasons after the full-text review**

Excluded studies after the full-text review for the following reasons: three studies were systematic reviews, (1-3) two studies did not mentioned the exposure diagnosis criteria (4, 5) two studies did not measure the outcome of interest, (6, 7) 23 studies that did not include TDIs group stratification (8-30) and one study had an adult sample only (31).

References

1. Borges TS, Vargas-Ferreira F, Kramer PF, Feldens CA. Impact of traumatic dental injuries on oral health-related quality of life of preschool children: A systematic review and meta-analysis. PLoS ONE [Electronic Resource]. 2017;12(2):e0172235.

2. Zaror C, Martiinez-Zapata MJ, Abarca J, Diaz J, Pardo Y, Pont A, et al. Impact of traumatic dental injuries on quality of life in preschoolers and schoolchildren: A systematic review and meta-analysis

3. Antunes LAA, Leao AT, Maia LC. The impact of dental trauma on quality of life of children and adolescents: a critical review and measurement instruments. Ciencia & Saude Coletiva. 2012;17(12):3417-24.

4. Castro FC, Raggio DP, Imparato JCP, Piovesan C, Bonini GC. Impact of oral problems on the quality of life of preschool children. Pesquisa Brasileira em Odontopediatria e Clinica Integrada. 2014;13(4):361-9.

5. Berger TD, Kenny DJ, Casas MJ, Barrett EJ, Lawrence HP. Effects of severe dentoalveolar trauma on the quality-of-life of children and parents. Dental Traumatology. 2009;25(5):462-9.

6. Wallace A, Rogers HJ, Zaitoun H, Rodd HD, Gilchrist F, Marshman Z. Traumatic dental injury research: on children or with children? Dental Traumatology. 2017;33(3):153-9.

7. Golai S, Nimbeni B, Patil SD, Baali P, Kumar H. Impact of Untreated Traumatic Injuries to Anterior Teeth on the Oral Health Related Quality of Life As Assessed By Video Based Smiling Patterns in Children. Journal of Clinical and Diagnostic Research JCDR. 2015;9(6):ZC16-9

8. Diaz S, Mondol M, Penate A, Puerta G, Bonecker M, Martins Paiva S, et al. Parental perceptions of impact of oral disorders on Colombian preschoolers' oral health-related quality of life. Acta Odontologica Latinoamericana. 2018;31(1):23-31.

9. Sardenberg F, Cavalcante-Leao BL, Todero SR, Ferreira FM, Rebellato NL, Fraiz FC. A population-based study on the impact of orofacial dysfunction on oral health-related quality of life among Brazilian schoolchildren. Acta Odontologica Scandinavica. 2017;75(3):173-8.

10. Perazzo MF, Gomes MC, Neves ET, Martins CC, Paiva SM, Costa E, et al. Oral problems and quality of life of preschool children: self-reports of children and perception of parents/caregivers. European Journal of Oral Sciences. 2017;125(4):272-9.

11. Ortiz FR, Ramadan YH, GonzÁLez RAB, Ardenghi TM. Factors associated with Oral Health-Related Quality of Life of preschool children in Southern Brazil. RGO - Revista Gaúcha de Odontologia. 2016;64(3):256-62.

12. Schuch HS, Costa Fdos S, Torriani DD, Demarco FF, Goettems ML. Oral health-related quality of life of schoolchildren: impact of clinical and psychosocial variables. International Journal of Paediatric Dentistry. 2015;25(5):358-65.

13. Ramos-Jorge J, Paiva SM, Tataounoff J, Pordeus IA, Marques LS, Ramos-Jorge ML. Impact of treated/untreated traumatic dental injuries on quality of life among Brazilian schoolchildren. Dental Traumatology. 2014;30(1):27-31.

14. Motamedi MRK, Behzadi A, Khodadad N, Zadeh AK, Nilchian F. Oral health and quality of life in children: A cross-sectional study. Dent Hypotheses. 2014;5(2):53-8.

15. Guedes RS, Piovesan C, Antunes JL, Mendes FM, Ardenghi TM. Assessing individual and neighborhood social factors in child oral health-related quality of life: a multilevel analysis. Quality of Life Research. 2014;23(9):2521-30.

16. El-Kalla IH, Shalan HM, Bakr RA. Impact of Dental Trauma on Quality of Life Among 11-14 Years Schoolchildren. Contemporary Clinical Dentistry. 2017;8(4):538-44.

17. Basavaraj P, Sunil MK, Nagarajappa R, Ashish S, Ramesh G. Correlation between oral health and Child-OIDP index in 12- and 15-year-old children from Modinagar, India. Asia-Pacific Journal of Public Health. 2014;26(4):390-400.

18. Scarpelli AC, Paiva SM, Viegas CM, Carvalho AC, Ferreira FM, Pordeus IA. Oral health-related quality of life among Brazilian preschool children. Community Dentistry & Oral Epidemiology. 2013;41(4):336-44.

19. Kramer PF, Feldens CA, Ferreira SH, Bervian J, Rodrigues PH, Peres MA. Exploring the impact of oral diseases and disorders on quality of life of preschool children. Community Dentistry & Oral Epidemiology. 2013;41(4):327-35.

20. Antunes LS, Debossan PF, Bohrer LS, Abreu FV, Quintanilha L, Antunes LAA. Impact of traumatic dental injury on the quality-of-life of children and adolescents: A case-control study. Acta Odontologica Scandinavica. 2013;71(5):1123-8.

21. Antunes LA, Antunes Ldos S, Luiz RR, Leao AT, Maia LC. Assessing the responsiveness of the Brazilian FIS to treatment for traumatic dental injury. Community Dentistry & Oral Epidemiology. 2013;41(6):551-7.

22. Paula JS, Leite IC, Almeida AB, Ambrosano GM, Pereira AC, Mialhe FL. The influence of oral health conditions, socioeconomic status and home environment factors on schoolchildren's self-perception of quality of life. Health & Quality of Life Outcomes. 2012;10:6.

23. Porritt JM, Rodd HD, Baker SR. Quality of life impacts following childhood dento-alveolar trauma. Dental Traumatology. 2011;27(1):2-9.

24. Abanto J, Carvalho TS, Mendes FM, Wanderley MT, Bonecker M, Raggio DP. Impact of oral diseases and disorders on oral health-related quality of life of preschool children. Community Dentistry & Oral Epidemiology. 2011;39(2):105-14.

25. Fakhruddin KS, Lawrence HP, Kenny DJ, Locker D. Impact of treated and untreated dental injuries on the quality of life of Ontario school children. Dental Traumatology. 2008;24(3):309-13.

26. Ramos-Jorge ML, Bosco VL, Peres MA, Nunes AC. The impact of treatment of dental trauma on the quality of life of adolescents - a case-control study in southern Brazil. Dental Traumatology. 2007;23(2):114-9.

27. Locker D. Disparities in oral health-related quality of life in a population of Canadian children. Community Dentistry & Oral Epidemiology. 2007;35(5):348-56.

28. Giannetti L, Murri A, Vecci F, Gatto R. Dental avulsion: therapeutic protocols and oral health-related quality of life. Eur J Paediatr Dent. 2007;8(2):69-75.

29. Cortes MI, Marcenes W, Sheiham A. Impact of traumatic injuries to the permanent teeth on the oral health-related quality of life in 12-14-year-old children. Community Dentistry & Oral Epidemiology. 2002;30(3):193-8.

30. Apaza-Ramos S, Torres-Ramos G, Blanco-Victorio DJ, Antezana-Vargas V, Montoya-Funegra J. Influencia de los factores sociodemográficos, familiares y el estado de la salud bucal en la calidad de vida de adolescentes peruanos. Revista Estomatológica Herediana. 2015;25(2):87-99

31. Johansson G, Ostberg AL. Oral health-related quality of life in Swedish young adults. International Journal of Qualitative Studies on Health and Well-Being. 2015;10.
