# Supplementary material for: Impact of uncomplicated traumatic dental injuries on the quality of life of children and adolescents: a systematic review and meta-analysis
Source: BMC Oral Health. 2019 Oct 22;19:224. doi: 10.1186/s12903-019-0916-0 (PMC6805369; doi:10.1186/s12903-019-0916-0)
Supplement: Supplementary file 4 — Additional file 4. Study characteristics and the association between uncomplicated TDI and OHRQoL in children and adolescents: Condensed tables that summarized the information extracted from each paper. [file 12903_2019_916_MOESM4_ESM.docx]

**Study characteristics and the association between uncomplicated TDI and OHRQoL in children and adolescents.**

Table 1: Case controls studies

| **Author and year, Title, country, type of population and sample size n/N (analysed sample/total sample)** | **TDI diagnosis criteria, Age exposure measured in years. How TDI was measured?** | **QOL measure, Age outcome was measured. Questionnaire used** | **Stratification, confounders/selection bias, reverse causation** | **Results** | **Findings by the authors** |
| --- | --- | --- | --- | --- | --- |
| **Firmino, Gomes et al. 2016 (69)**,  Impact of oral health problems on the quality of life of preschool children: a case–control study,  Brazil,  population-based,  n/N= 830/845 | Andreasen classification,  Age – 3-5,  Clinical examination | OHRQoL  Age – 3-5,  ECOHIS | Matched for sex, age and monthly household income | \| TDI’s impact in QOL \| \| \| \| --- \| --- \| --- \| \| Uncomplicated TDI \| Quality of Life Impacted  (Unadjusted OR) \| 95 % CI (P-value) \| \| Absent \| REF \| REF \| \| Present \| 1.57 \| 0.92-2.64 (0.0741) \| | Pre-schoolers with uncomplicated TDI have greater odds to have their oral health related quality of life impacted than children without TDIs. |
| **Vieira-Andrade, Siqueira et al. 2015 (67)**, Impact of traumatic dental injury on the quality of life of young children: a case–control study,  Brazil,  population-based,  n/N= 335/335 | Andreasen classification,  Age – 3-5  Clinical examination | OHRQoL ,  Age – 3-5,  ECOHIS | Adjusted for: dental caries and malocclusion.  Matched for age, gender, type of preschool and monthly household income. | \| TDI’s impact in QOL \| \| \| \| --- \| --- \| --- \| \| Uncomplicated TDI \| Quality of Life Impacted  (Adjusted OR) \| 95 % CI (P-value) \| \| Absent \| REF \| REF \| \| Present \| 1.05 \| 0.54-1.99 (0.8657) \| | Uncomplicated TDI had no impact on the quality of life of preschool children. |
| **Bendo, Paiva et al. 2014 (68),**  Oral health-related quality of life and traumatic dental injuries in Brazilian adolescents,  Brazil,  population-based,  n/N= 1215/1215 | Andreasen classification,  Age – 11-14  Clinical examination | OHRQol,  Age – 11-14  CPQ11–14 | Adjusted for: dental caries and malocclusion.  Matched for gender and type of school | \| TDI’s impact in QOL \| \| \| \| --- \| --- \| --- \| \| Uncomplicated TDI \| Quality of Life Impacted  (Adjusted OR) \| 95 % CI (P-value) \| \| Absent \| REF \| REF \| \| Present \| 0.64 \| 0.38- 1.06 (0.0733) \| | Mild TDI and were not associated with negative impact on OHRQoL of adolescents. |

**Table 2:** Cross-sectional studies

| **Author and year, Title, country, type of population and sample size n/N (analysed sample/total sample)** | **TDI diagnosis criteria, Age exposure measured in years. How TDI was measured?** | **QOL measure, Age outcome was measured. Questionnaire used** | **Stratification, confounders/selection bias, reverse causation** | **Results** | **Findings by the authors** |
| --- | --- | --- | --- | --- | --- |
| **(Soares, Barasuol et al. 2018) (87)**,  The impact of crown fracture in the permanent dentition on children’s quality of life,  Brazil  population-based,  n/N= 1589/1671 | Andreasen classification  Age – 8-10  Clinical examination | OHRQoL  Age – 8-10  CPQ8-10 | Adjusted for: socio-demographic characteristics (monthly family income and caregivers’ schooling), characteristics of the child (gender, age) and clinical oral conditions (DMFT, DAI, overjet and TDI) | \| TDI’s impact in QOL \| \| \| \| --- \| --- \| --- \| \| TDI \| Quality of Life Impacted PR \| 95 % CI (p-value) \| \| Without fracture \| REF \| REF \| \| Enamel Fracture \| 0.9 \| 0.72-1.08 (0.254) \| \| Enamel and dentin Fracture \| 1.35 \| 1.07-1.70 (0.009) \| | Enamel fracture had no significant impact on children’ quality of life, while enamel-dentin fracture had a 35% higher prevalence of impact on the OHRQoL compared to those without TDI. |
| **Silva-Oliveira, Goursand et al. 2018 (2)** Traumatic dental injuries in Brazilian children and oral health related quality of life,  Brazil,  Population-based,  n/N= 588/633 | Andreasen classification,  Age – 12,  Clinical examination | OHRQoL,  Age – 12,  CPQ11-14 | Adjusted for: overjet, gender, mother’s schooling, monthly household income, and type of school. | \| TDI’s impact in QOL \| \| \| \| --- \| --- \| --- \| \| Uncomplicated TDI \| Quality of Life Impacted OR \| 95 % CI (p-value) \| \| Absence \| REF \| REF \| \| present \| Adjusted OR 1.47 \| 1.004 -2.17 (0.0382) \| | Uncomplicated TDI associated with impact in adolescent quality of life. |
| **Martins, Sardenberg et al. 2018 (70),**  Dental caries are more likely to impact on children’s quality of life than malocclusion or traumatic dental injuries, Brazil,  population-based,  n/N= 1,204/1,439 | Andreasen classification,  Age – 8 -10,  Clinical examination | OHRQoL,  Age – 8 -10,  CPQ8-10 | Adjusted for oral conditions. | \| TDI’s impact in QOL \| \| \| \| --- \| --- \| --- \| \| Uncomplicated TDI \| Quality of Life Impacted  OR \| 95 % CI (p-value) \| \| Absence \| REF \| REF \| \| present \| 1.04 \| 0.83-1.30 (0.756) \| | The children with TDI did not show statistically significant difference on the CPQ8-10 when compared with those without oral conditions, probably because enamel fracture was the major finding. |

**Table 2:** (continued)

| **Author and year, Title, country, type of population and sample size n/N (analysed sample/total sample)** | **TDI diagnosis criteria, Age exposure measured in years. How TDI was measured?** | **QOL measure, Age outcome was measured. Questionnaire used** | **Stratification, confounders/selection bias, reverse causation** | **Results** | **Findings by the authors** |
| --- | --- | --- | --- | --- | --- |
| **Ramos-Jorge, Sa-Pinto et al. 2017 (30),**  Effect of dark discolouration and enamel/dentine fracture on the oral health-related quality of life of pre-schoolers,  Brazil,  preschool-based,  n/N= 391/459 | Andreasen classification,  Age – 3-5,  Clinical examination | OHRQoL,  Age – 3-5  ECOHIS | Adjusted for: characteristics of the child (gender and age), home, monthly household income, type of pre-school (public or private) and family provider) and clinical oral conditions (TDI and untreated dental caries) | \| TDI’s impact in QOL \| \| \| \| --- \| --- \| --- \| \| TDI \| Quality of Life Impacted PR \| 95 % CI (p-value) \| \| Without fracture \| REF \| REF \| \| Enamel Fracture \| 1.30 \| 0.79–2.16 (0.296) \| \| Enamel and dentin Fracture \| 1.89 \| 1.22–2.92 (<0.001) \| | Enamel–dentine fracture without pulp exposure were associated with a negative impact on the QoL of pre-schoolers. |
| **Neves, Perazzo et al. 2017 (71)**  Perception of parents and self-reports of children regarding the impact of traumatic dental injury on quality of life,  Brazil,  school-based  n/N= 769/769 | Andreasen classification,  Age – 5,  Clinical examination  by two dentists | OHRQoL,  Age – 5,  SOHO-5 | Adjusted for: child’s gender, schooling of parent/caregiver,  monthly household income, number of residents in home, toothache, TDI, type of TDI, and number of  teeth with TDI | \| TDI’s impact in QOL \| \| \| \| --- \| --- \| --- \| \| Uncomplicated TDI \| Quality of Life Impacted Adjusted PR \| 95% CI (p-value) \| \| Absence \| REF \| REF \| \| Presence \| 0.66 \| 0.44-0.98 (0.044) \| | The occurrence of non-complicated TDI was seen as a protective factor for OHRQoL according to the reports of the children. |
| **Gonçalves, Dias et al. 2017 (72)**,  Impact of dental trauma and esthetic impairment on the quality of life of preschool children,  Brazil,  school-based,  n/N= 192/192 | Andreasen classification,  Age – 2-5,  Clinical examination | OHRQoL,  Age – 2-5,  ECOHIS | Potential confounding trauma and gender, age, quality of life and esthetic impairment. | \| TDI’s impact in QOL \| \| \| \| --- \| --- \| --- \| \| Uncomplicated TDI \| Quality of Life Impacted RR \| 95% CI (p-value) \| \| Absence \| REF \| REF \| \| Presence \| 1.48 \| 1.20-1.83 (0.0107) \| | The presence of uncomplicated TDIs did not inﬂuence negatively on children’s quality of life, |

**Table 2:** (continued)

| **Author and year, Title, country, type of population and sample size n/N (analysed sample/total sample)** | **TDI diagnosis criteria, Age exposure measured in years. How TDI was measured?** | **QOL measure, Age outcome was measured. Questionnaire used** | **Stratification, confounders/selection bias, reverse causation** | **Results** | **Findings by the authors** |
| --- | --- | --- | --- | --- | --- |
| **Gomes, Perazzo et al. 2017 (73),**  Oral Problems and Self-Confidence in Preschool Children,  Brazil,  school-based,  n/N= 769/769 | Andreasen classification,  Age – 5  Clinical examination | OHRQoL  Age – 5  SOHO-5 | Not adjusted for confounders. | \| TDI’s impact in QOL \| \| \| \| --- \| --- \| --- \| \| Uncomplicated TDI \| Unadjusted Prevalence ratio \| 95 % CI (p-value) \| \| Absent \| REF \| REF \| \| Presence \| 0.51 \| 0.23-1.11 (0.091) \| | Uncomplicated TDIs did not affected quality of life of preschool children |
| **Bomfim, Herrera et al. 2017 (88)**,  Oral health-related quality of life and risk factors associated with traumatic dental injuries in Brazilian children: A multilevel approach,  Brazil,  population-based,  n/N= 7328/7328 | Five oral health outcomes based on the original data set were used  as dependent variables: trauma 12, trauma 11, trauma 21, trauma 22,  trauma 31, trauma 32, trauma 41 and trauma 42,  Age – 12,  Clinical examination | OHRQoL,  Age – 12,  Oral Impact on Daily Performance (OIDP) questionnaire | Adjusted for: gender, skin colour, education and family income.  Education, a continuous variable ranging from 0 to 15, was assessed by estimating the number of years of complete education without retention. Self-reported skin colour was classified as white and non-white (black, mulatto and others). Monthly family income | \| TDI’s impact in QOL \| \| \| \| --- \| --- \| --- \| \| Enamel Fracture \| Shame Adjusted OR \| 95% CI (p-value) \| \| Absent \| REF \| REF \| \| Present \| 1.27 \| 1:05-1:53 (<0.05) \|  \| Dentin/enamel fractures \| Shame Adjusted OR \| 95% CI (p-value) \|  \| \| --- \| --- \| --- \| --- \| \| Absent \| REF \| REF \|  \| \| Present \| 1.89 \| 1.30-2.74 (<0.05) \|  \| | Enamel fractures were risk factors for feelings of shame among children, whereas dentin/enamel fractures had higher probability of children having dissatisfaction with their teeth or for feeling embarrassed at smiling and messing up with the study. |
| **Pulache, Abanto et al. 2016 (74)**,  Exploring the association between oral health problems and oral health-related quality of life in Peruvian 11- to 14-year-old children,  Peru,  school-based,  n/N= 473/513 | Andreasen classification,  Age – 11-14,  Clinical examination | OHRQol,  Age – 11-14  CPQ11–14 | Adjusted for: Child age, Child gender, Dental caries experience, TDI, Malocclusion, Tooth discoloration  Possible observer bias | \| TDI’s impact in QOL \| \| \| \| --- \| --- \| --- \| \| Uncomplicated TDI \| Adjusted RR \| 95 5 CI (P-value) \| \| Absent \| REF \| REF \| \| Present \| 1.04 \| 1- 1.09 (0.05) \| | Presence of uncomplicated TDI doesn’t have an impact on children’s OHRQoL |

**Table 2:** (continued)

| **Author and year, Title, country, type of population and sample size n/N (analysed sample/total sample)** | **TDI diagnosis criteria, Age exposure measured in years. How TDI was measured?** | **QOL measure, Age outcome was measured. Questionnaire used** | **Stratification, confounders/selection bias, reverse causation** | **Results** | **Findings by the authors** |
| --- | --- | --- | --- | --- | --- |
| **Feldens, Day et al. 2016 (89),**  Enamel fracture in the primary dentition has no impact on children’s quality of life: implications for clinicians and researchers,  Brazil,  population-based  n/N= 1683 /1683 | Andreasen classification,  Age – 1-5,  Clinical examination | OHRQoL  Age – 1-5,  ECOHIS | Adjusted for: age, dental caries, and malocclusion, Attendance at a dental professional and number of teeth. | \| TDI’s impact in QOL \| \| \| \| --- \| --- \| --- \| \| TDI \| Quality of Life Impacted Adjusted PR \| 95 5 CI (P-value) \| \| Absent \| REF \| REF \| \| Enamel fracture \| 1.10 \| 0.62–1.93 (0.753) \| \| Other TDI \| 1.87 \| 1.39–2.52 (<0.001) \| | Enamel fractures have no significant impact on young children’s quality of life. |
| **Freire-Maia, Auad et al. 2015 (84)**,  Oral Health-Related Quality of Life and Traumatic Dental Injuries in Young Permanent Incisors in Brazilian Schoolchildren: A Multilevel Approach,  Brazil,  school-based,  n/N= 1201/1,201 | Andreasen classification,  Age – 8-10,  Clinical examination | OHRQoL ,  Age – 8-10,  CPQ8–10 | Adjusted for: were gender, age,  TDI, dental caries, anterior open bite, anterior maxillary overjet, median diastema, upper anterior crowding and lower anterior crowding, family income, residents in home, parents/caregivers’ level of education, type of school and SVI. | \| TDI’s impact in QOL \| \| \| \| --- \| --- \| --- \| \| TDI \| Quality of Life Impacted (Adjusted OR) \| 95 5 CI (P-value) \| \| Without TDI/mild trauma \| REF \| REF \| \| Severe trauma \| 2.54 \| 1.21–5.31 (0.014) \| | Severe trauma was significantly associated with negative impact on overall quality of Life. |

**Table 2:** (continued)

| **Author and year, Title, country, type of population and sample size n/N (analysed sample/total sample)** | **TDI diagnosis criteria, Age exposure measured in years. How TDI was measured?** | **QOL measure, Age outcome was measured. Questionnaire used** | **Stratification, confounders/selection bias, reverse causation** | **Results** | **Findings by the authors** |
| --- | --- | --- | --- | --- | --- |
| **Abanto, Tello et al. 2015 (75)**,  Impact of traumatic dental injuries and malocclusions onquality of life of preschool children,  Brazil,  population-based,  n/N= 1215/1215 | Glendor classification,  Age 1-4,  Clinical examination | OHRQoL,  Age 1-4,  ECOHIS | Adjusted for: dental caries, malocclusions, and socio-demographic conditions. | \| TDI’s impact in QOL \| \| \| \| --- \| --- \| --- \| \| TDI \| Quality of life impacted (adjusted PR) \| 95 % CI (P-value) \| \| Absent \| REF \| REF \| \| Uncomplicated  injuries \| 0.75 \| 0.55–1.03 (0.068) \| | The presence of uncomplicated TDI is not associated with worse OHRQoL of Brazilian preschool children. |
| **Viegas, Paiva et al. 2014 (31)**,  Influence of traumatic dental injury on quality of life of Brazilian preschool children and their families,  Brazil,  school-based,  n/N= 1632/1632 | Andreasen classification,  Age – 5-6,  Clinical examination | OHRQoL,  Age – 5-6,  ECOHIS | Adjusted for: dental caries experience | \| TDI’s impact in QOL \| \| \| \| --- \| --- \| --- \| \| Uncomplicated TDI \| Quality of life impacted OR \| 95 % CI (p-value) \| \| Absence \| REF \| REF \| \| Presence \| 0.91 \| 0.73-1.13 (0.3799) \| | The presence of uncomplicated TDI in Brazilian preschool children has no impact on quality of life of the children or their families. |
| **Gomes, Pinto-Sarmento et al. 2014 (76)**, Impact of oral health conditions on the quality of life of preschool children and their families: A cross-sectional study,  Brazil,  preschool-based,  n/N= 834/864 | Andreasen classification,  Age – 3-5,  Clinical examination | OHRQoL,  Age – 3-5,  ECOHIS | Adjusted for: sex, age, type of preschool, mother’s schooling, monthly household income, parent’s/guardian’s age, | \| TDI’s impact in QOL \| \| \| \| --- \| --- \| --- \| \| Uncomplicated TDI \| Quality of life impacted OR \| 95 % CI (p-value) \| \| Absence \| REF \| REF \| \| present \| adjusted OR 0.98 \| 0.69- 1.38 (0.9002) \| | Negative quality of life impact in children occurs in more serious TDI cases. |

**Table 2:** (continued)

| **Author and year, Title, country, type of population and sample size n/N (analysed sample/total sample)** | **TDI diagnosis criteria, Age exposure measured in years. How TDI was measured?** | **QOL measure, Age outcome was measured. Questionnaire used** | **Stratification, confounders/selection bias, reverse causation** | **Results** | **Findings by the authors** |
| --- | --- | --- | --- | --- | --- |
| **Abanto, Tsakos et al. 2014 (77)**,  Impact of dental caries and trauma on quality of life among 5- to 6-year-old children perceptions of parents and children,  Brazil,  Dental school-based,  n/N= 335/394 | Glendor classification,  Age – 5-6  Clinical examination | OHRQoL  Age – 5-6  SOHO-5 | Adjusted for: caries and sociodemographic conditions (child’s gender, child’s age, family income) | \| TDI’s impact in QOL \| \| \| \| --- \| --- \| --- \| \| TDI \| Quality of life impacted RR \| 95 % CI (P-value) \| \| Absent \| REF \| REF \| \| Uncomplicated  injuries \| 0.69 \| 0.51–0.94 (0.019) \| | TDIs are not associated with worse OHRQoL of 5- to 6-year-old children in terms of perceptions of both children and their parents. |
| **Siqueira, Firmino et al. 2013 (78)**,  Impact of Traumatic Dental Injury on the Quality of Life of Brazilian Preschool Children,  Brazil,  population-based,  n/N= 814/864 | Andreasen classification,  Age – 3-5,  Clinical examination | OHRQol  Age – 3-5,  ECOHIS | Adjusted for: dental caries and malocclusion | \| TDI’s impact in QOL \| \| \| \| --- \| --- \| --- \| \| Uncomplicated TDI \| Quality of life impacted OR \| 95 % CI (p-value) \| \| Absence \| REF \| REF \| \| Presence \| 1.02 \| 0.74 1.41 (0.8974) \| | No association between TDI and OHRQoL. |
| **Dame-Teixeira, Alves et al. 2013 (85)**,  Traumatic dental injury with treatment needs negatively affects the quality of life of Brazilian schoolchildren,  Brazil,  school-based,  n/N=1528/1837 | O’Brien classification,  Age – 14,  Clinical examination | OHRQoL,  Age – 14,  CPQ11–14 | Adjusted for: gender, socioeconomic status, malocclusion, and dental caries | \| TDI’s impact in QOL \| \| \| \| --- \| --- \| --- \| \| TDI \| Quality of Life impacted RR \| 95 % CI (p-value) \| \| No TDI/No treatment  need \| REF \| REF \| \| Treatment need \| 1.09 \| 0.92–1.29(P < 0.05) \| | 97% of the total TDIs in the study were uncomplicated TDI. TDI. Those with treatment needs had a negative effect in the OHRQoL in this population of 12-year-old schoolchildren |

**Table 2:** (continued)

| **Author and year, Title, country, type of population and sample size n/N (analysed sample/total sample)** | **TDI diagnosis criteria, Age exposure measured in years. How TDI was measured?** | **QOL measure, Age outcome was measured. Questionnaire used** | **Stratification, confounders/selection bias, reverse causation** | **Results** | **Findings by the authors** |
| --- | --- | --- | --- | --- | --- |
| **Viegas, Scarpelli et al. 2012 (79)**,  Impact of Traumatic Dental Injury on Quality of Life Among Brazilian Preschool Children and Their Families,  Brazil,  preschool-based,  n/N= 388 /413 | Andreasen classification,  Age – 5  Clinical examination | OHRQoL  Age –5,  ECOHIS | Adjusted for: clinical factors (malocclusion, dental caries, and developmental defects of enamel) and socioeconomic factors (household income and social vulnerability index) | \| TDI’s impact in QOL \| \| \| \| --- \| --- \| --- \| \| Uncomplicated TDI \| Quality of Life impacted adjusted RR \| 95 % CI (p-value) \| \| No \| REF \| REF \| \| Yes \| 1.15 \| 1.15-1.71 (0.001) \| | Quality of life of the children and their families was not inﬂuenced by the presence of dental trauma |
| **Traebert, de Lacerda et al. 2012 (86)**,  Impact of traumatic dental injuries on the quality of life of schoolchildren,  Brazil,  population-based,  n/N= 403/409 | WHO criteria,  Age – 11-14,  Clinical examination | OHRQoL  Age – 11-14,  CPQ11–14 | Adjusted for: Gender, age, mother’s education level, father’s education level, whether fathers were currently working, the presence of TDI, caries experience in the anterior dentition, and presence of malocclusion | \| TDI’s impact in QOL \| \| \| \| --- \| --- \| --- \| \| TDI \| Quality of Life impacted adjusted PR \| 95 % CI (p-value) \| \| Absence \| REF \| REF \| \| Presence \| 1.79 \| 1.16–2.76 (0.008) \| | Traumatic dental injuries appear to affect schoolchildren’s OHRQoL. |
| **Piovesan, Abella et al. 2011 (80)**,  Child Oral Health-related Quality of Life and Socioeconomic Factors Associated with Traumatic Dental Injuries in Schoolchildren,  Brazil,  school-based,  n/N= 713/ 792 | O’Brien classification,  Age – 12,  Clinical examination | OHRQoL  Age – 12,  CPQ11–14 | Adjusted for: gender, parents’ educational level, household income, overjet and lip coverage. | \| TDI’s impact in QOL \| \| \| \| --- \| --- \| --- \| \| TDI \| Quality of Life impacted adjusted RR \| 95 % CI (p-value) \| \| Absence \| REF \| REF \| \| Presence \| 1.01 \| 0.86 – 1.19 (0.84) \| | TDIs are not related to children quality of life. Taken together, the low prevalence and severity of TDI (98.9% uncomplicated TDI) reported in this study could have inﬂuenced the lack of association between dental trauma and OHRQoL |

**Table 2:** (continued)

| **Author and year, Title, country, type of population and sample size n/N (analysed sample/total sample)** | **TDI diagnosis criteria, Age exposure measured in years. How TDI was measured?** | **QOL measure, Age outcome was measured. Questionnaire used** | **Stratification, confounders/selection bias, reverse causation** | **Results** | **Findings by the authors** |
| --- | --- | --- | --- | --- | --- |
| **Aldrigui, Abanto et al. 2011 (81)**,  Impact of traumatic dental injuries and malocclusions on quality of life of young children,  Brazil,  preschool-based,  n/N= 260/305 | Andreasen classification,  Age – 2-5,  Clinical examination | OHRQoL  Age – 2-5  ECOHIS | Adjusted for: anterior malocclusions traits, caries, Age. | \| TDI’s impact in QOL \| \| \| \| --- \| --- \| --- \| \| Uncomplicated TDI \| Quality of Life impacted adjusted RR \| 95 % CI (p-value) \| \| Absence \| REF \| REF \| \| Presence \| 0.89 \| 0.66 - 1.20 (0.441) \| | Uncomplicated traumatic dental injuries have no negative impact on the OHRQoL of preschool children and their parents. |
| **Bendo, Paiva et al. 2010 (83)**,  Association between treated/untreated traumatic dental injuries and impact on quality of life of Brazilian schoolchildren,  Brazil,  school-based,  n/N= 1612 /1870 | Andreasen classification,  Age – 11-14,  Clinical examination | OHRQoL  Age – 11-14,  CPQ11-14 | Not adjusted. | \| TDI’s impact in QOL \| \| \| \| --- \| --- \| --- \| \| TDI \| Quality of Life Impacted (Unadjusted PR) \| 95 % CI (p-value) \| \| Absence \| REF \| REF \| \| Untreated TDI \| 0.6 \| 0.2-1.6 (0.368) \| | Untreated TDI (96% of the sample had uncomplicated TDI) was not associated with oral symptoms, functional limitations or emotional wellbeing |
| **Piovesan, Antunes et al. 2010 (82)**,  Impact of socioeconomic and clinical factors on child oral health-related quality of life (COHRQoL),  Brazil,  n/N= 713/ 792 | O’Brien classification,  Age – 12,  Clinical examination | OHRQoL  Age – 12,  CPQ11–14 | none | \| TDI’s impact in QOL \| \| \| \| --- \| --- \| --- \| \| TDI \| Quality of life impacted (Unadjusted RR) \| 95 % CI (p-value) \| \| Absence \| REF \| REF \| \| Presence \| 1.00 \| 0.86–1.18 (0.91) \| | Dental trauma was not associated with quality of life in adolescents |
